# Association of alcohol dehydrogenase and aldehyde dehydrogenase Polymorphism with Spontaneous Deep Intracerebral Haemorrhage in the Taiwan population

**DOI:** 10.1038/s41598-020-60567-5

**Published:** 2020-02-27

**Authors:** Yu-Hua Huang, Kuo-Hsuan Chang, Yun-Shien Lee, Chiung-Mei Chen, Yi-Chun Chen

**Affiliations:** 1 Department of Neurology, Chang Gung Memorial Hospital Linkou Medical Center and College of Medicine, Chang-Gung University, Taoyuan, 333 Taiwan; 2 Department of Biotechnology, Ming Chuan University, Taoyuan, 333 Taiwan; 3 Genomic Medicine Research Core Laboratory, Chang Gung Memorial Hospital, Taoyuan, 333 Taiwan

**Keywords:** Genetics research, Stroke

## Abstract

Alcohol dehydrogenase (*ADH*) and aldehyde dehydrogenase (*ALDH*) encode essential alcohol-metabolizing enzymes. While alcohol use is associated with spontaneously deep intracerebral haemorrhage (SDICH), particularly in males, the activities and genetic variants of *ADH* and *ALDH* may affect SDICH development. This case-control study was conducted to identify the interaction of alcohol use and SDICH with five single-nucleotide polymorphisms (SNPs): *ADH1B* rs1229984, *ADH1C* rs2241894, *ALDH2* rs671, *ALDH2* rs886205, and *ALDH2* rs4648328. We enrolled 208 patients with SDICH and 244 healthy controls in a Taiwanese population. *ALDH2* rs671 was significantly associated with SDICH in the dominant (*P* < 0.001) and additive models (*P* = 0.007). *ALDH2* rs4648328 was borderline significantly associated with SDICH in the recessive (*P* = 0.024) or additive models (*P* = 0.030). In alcohol-using patients, the *ALDH2* rs671 GG genotype was associated with SDICH risk compared to the GA+AA genotype (*P* = 0.010). *ADH1B* rs1229984, *ADH1C* rs2241894, and *ALDH2* rs886205 did not demonstrate association with SDICH. Thus, the *ALDH2* rs671 GG genotype is a risk factor for SDICH. Because the genetic distributions of *ALDH2* rs671 exhibited strong ethnic heterogeneity, further studies in different populations are needed to validate these findings.

## Introduction

Primary intracerebral haemorrhage (ICH), accounting for 22–35% of all cases of stroke in Asian populations^1^, is the most devastating stroke subtype with high rates of death and long-term disability in adults^2,3^. Asian populations have higher incidences of primary ICH than Caucasians^2^. Sixty to eighty percent of primary ICH cases occur at the non-lobar region, including the basal ganglia, thalamus, brain stem, and cerebellum, and are also known as spontaneously deep intracerebral haemorrhage (SDICH)^4^. Numerous factors, such as hypertension and alcohol use, have been proposed to contribute to SDICH development^5,6^.

Alcohol use was associated with an increased ICH risk^7^. Alcohol is primarily metabolized by alcohol dehydrogenase (ADH) and aldehyde dehydrogenase (*ALDH*)^8^. The metabolism of alcohol produces acetaldehyde, acetate, and reactive oxygen species (ROS). Excessively produced acetaldehyde and ROS, which are highly reactive and toxic by-products, are distributed throughout cell membranes and interact with certain proteins, affecting cell function and leading to organs damage. The accumulation of acetaldehyde causes oxidative damage, excessive autophagy, decreased myofilament calcium sensitivity, and impaired endoplasmic reticulum calcium-ATPase function^9^. Acetate metabolism involved in lipid biosynthesis in the mitochondria of brain tissues^10^. In animal models, toxic aldehydes enlarged the cerebral ischaemia-induced infarct area and increased oxidative stress^11,12^. Deceased enzymatic activity of ALDH, a condition which impairs the degradation of acetaldehyde, could be associated with higher alcohol intoxication among East Asians compared to Caucasians^13^. Previous studies suggested an association between genetic variants in the alcohol metabolism pathway and vascular diseases^14^. Individuals with the *ALDH2* rs671 A allele have higher prevalence of hypertension, cardiovascular risk factors, and cerebral infarction^15^. Polymorphisms in *ALDH2* rs671 are associated with coronary artery disease (CAD) in Chinese patients with hypertension^16^. In the male Japanese population, the *ALDH2* rs671 GG genotype is associated with cerebral lacunar infarcts^17^. In contrast, presence of the *ALDH2* rs671 A allele could be a risk factor for cerebral infarction in Han-Chinese population^15^. A reduction in ALDH2 activity may interfere with endothelium angiogenesis and is associated with cerebral amyloid angiopathy^18^.

However, the association between the genetic variants involved in alcohol metabolism and SDICH remains unclear. Here, we conducted a case-control study to investigate the associations of genetic variants in *ADH* and *ALDH*, including *ADH1B* rs1229984, *ADH1C* rs2241894, *ALDH2* rs671, *ALDH2* rs886205, and *ALDH2* rs4648328, and SDICH in a Taiwanese population.

## Results

### Patient characteristics

Among the 208 cases with SDICH and 244 controls, the percentage of men (67.8%) and those with hypertension (89.9%) were significantly higher in the SDICH group compared to in control group (men: 52.5%, *P* = 0.001; hypertension: 42.4%, *P* < 0.001, Table 1). More patients with SDICH had were exposed to alcohol (32.2%) or smoking (44.2%) compared to controls (alcohol use: 14.8%, *P* < 0.001; smoking: 19.3%, *P* < 0.001). The levels of total cholesterol (184.6 ± 38.9 mg/dL) in patients with SDICH were lower compared to in controls (total cholesterol: 200.2 ± 42.9 mg/dL, *P* = 0.001). Alcohol use (SDICH vs controls: 46.1% vs 26.0%, *P* = 0.001) and smoking (SDICH vs controls: 63.8% vs 35.4%, *P*  < 0.001) were more frequently observed in the male patients with SDICH (Table 1).

**Table 1:** Demographic data of the study population.

	All (N = 452)		*P*-Value	Male (N = 269)		*P*-Value	Female (N = 183)		*P*-Value
	SDICH	Control		SDICH	Control		SDICH	Control	
	(N = 208)	(N = 244)		(N = 141)	(N = 128)		(N = 67)	(N = 116)	
Age (years)	57.4 ± 10.5	60.0 ± 10.5	0.012	55.3 ± 10.2	59.5 ± 10.6	0.002	61.9 ± 9.9	60.5 ± 10.5	0.378
Male gender	141 (67.8%)	128 (52.5%)	0.001						
Hypertension	187 (89.9%)	103 (42.4%)	< 0.001	125 (88.7%)	57 (44.8%)	< 0.001	62 (92.5%)	46 (39.6%)	< 0.001
Diabetes mellitus	39 (18.8%)	41 (16.9%)	0.602	22 (15.6%)	21 (16.5%)	0.835	17 (25.4%)	20 (17.2%)	0.189
Alcohol use	67 (32.2%)	36 (14.8%)	< 0.001	65 (46.1%)	33 (26.0%)	0.001	2 (3%)	3 (2.6%)	0.873
Smoke	92 (44.2%)	47 (19.3%)	< 0.001	90 (63.8%)	45 (35.4%)	< 0.001	2 (3%)	2 (1.7%)	0.579
Total cholesterol (mg/dL)	184.6 ± 38.9	200.2 ± 42.9	0.001	184.0 ± 35.2	193.8 ± 46.6	0.076	185.9 ± 46.0	208.3 ± 36.4	0.003

Data are expressed as number, percentage, or mean ± SD.

Comparisons between controls and ICH group were analysed by Chi-square test or *t*-test where appropriate.

### Genotype frequency and association analysis of controls and patients with SDICH

All single-nucleotide polymorphisms (SNPs) were in Hardy-Weinberg equilibrium in the case and control groups according to the standard χ^2^ test at a significance level of 0.05. The genotype frequencies of the analysed SNPs in the case and control groups are shown in Table 2. *ALDH2* rs671 was significantly associated with SDICH in the dominant model (OR = 0.5, 95% CI: 0.4–0.8, *P* < 0.001) and additive model (OR = 0.7, 95% CI: 0.5–0.9, *P* = 0.007). The significance remained after adjusting for sex and age in the dominant model (OR = 0.6, 95% CI: 0.4–0.8, *P = *0.003) and borderline in the additive model (OR = 1.5, 95% CI: 1.1–2.0, *P = *0.015). However, these associations did not remain after further adjusting for hypertension and alcohol use. *ALDH2* rs4648328 could be associated with SDICH in the recessive model (OR = 2.4, 95% CI: 1.1–5.1, *P* = 0.024) and additive model (OR = 1.4, 95% CI: 1.0–1.9, *P* = 0.030) with boardline significance. These associations were not detected after Bonferroni correction and multivariate adjustment. The genotypic frequencies of other genetic variants were similar between the SDICH and controls.

**Table 2:** Genotypes of the SNPs and their associations with risk of spontaneously deep intracerebral haemorrhage (SDICH).

Gene	SNP ID	Genotype	SDICH (%)	Control (%)	Model 1	Model 2	Model 3
					OR (95% CI), *P* value	OR (95% CI), *P* value	*P* value
ALDH2	**rs671**	GG	133 (63.9)	118 (48.4)			
		GA	59 (28.4)	105 (43.0)			
		AA	16 (7.7)	21 (8.6)			
		Dominant model			0.5 (0.4–0.8), < 0.001	0.6 (0.4–0.8), 0.003	0.231
		Additive model			0.7 (0.5–0.9), 0.007	1.5 (1.1–2.0), 0.015	0.528
		Recessive model			0.724	0.685	0.496
	**rs4648328**	CC	111 (53.4)	148 (60.6)			
		CT	76 (36.5)	85 (34.8)			
		TT	21 (10.1)	11 (4.5)			
		Dominant model			0.119	0.221	0.725
		Additive model			1.4 (1.0–1.9),0.030	0.072	0.552
		Recessive model			2.4 (1.1–5.1),0.024	0.046	0.448
	**rs886205**	GG	154 (74.0)	179 (73.4)			
		GA	52 (25.0)	59 (24.2)			
		AA	2 (1.0)	6 (2.4)			
		Dominant model			0.871	0.852	0.777
		Additive model			0.636	0.617	0.524
		Recessive model			0.246	0.237	0.173
ADH	**rs1229984**	TT	112 (53.8)	129 (52.9)			
		TC	80 (38.5)	105 (43.0)			
		CC	16 (7.7)	10 (4.1)			
		Dominant model			0.836	0.816	0.817
		Additive model			0.646	0.662	0.607
		Recessive model			0.107	0.107	0.075
	**rs2241894**	CC	103 (49.5)	125 (51.2)			
		CT	92 (44.2)	101 (41.4)			
		TT	13 (6.3)	18 (7.4)			
		Dominant model			0.717	0.724	0.376
		Additive model			0.920	0.896	0.347
		Recessive model			0.637	0.701	0.589

SDICH: spontaneous deep intracerebral haemorrhage, OR: Odds ratio, CI: confidence interval.

Analysis were performed by logistic regression under dominant, additive and recessive genetic models.

Model 1: Crude logistic regression.

Model 2: Multivariable logistic regression, adjust sex, age.

Model 3: Multivariable logistic regression, adjust sex, age, HTN and alcohol.

*P*-value with Bonferroni correction for significance was 0.01.

The minor allele frequencies (MAFs) of the analysed SNPs in the case and control groups are shown in Table 3. The MAF of *ALDH2* rs671 (21.9%) in the SDICH group was significantly lower compared to controls (30.1%, odds ratio (OR) = 0.7, 95% confidence interval (CI): 0.5–0.9, *P* = 0.005). The MAFs of the other SNPs were similar between the SDICH and control groups.

**Table 3:** Allele frequencies of SNPs and their associations with risk of spontaneously deep intracerebral haemorrhage (SDICH).

Gene	SNP ID	All cases MAF	MAF		Model 1	Model 2	Model 3
			SDICH (%)	Control (%)	OR (95% CI), *P* value	*P* value	*P* value
ALDH2	**rs671**	A/0.263	0.219	0.301	0.7 (0.5–0.9), 0.005	0.012	0.523
	**rs4648328**	T/0.249	0.284	0.219	1.4 (1.0–1.9), 0.026	0.065	0.543
	**rs886205**	A/0.141	0.135	0.146	0.640	0.620	0.528
ADH	**rs1229984**	C/0.262	0.269	0.256	0.655	0.671	0.617
	**rs2241894**	T/0.282	0.284	0.281	0.923	0.899	0.365

SDICH: spontaneous deep intracerebral haemorrhage, OR: Odds ratio, CI: confidence interval, MAF: minor allele frequency.

Analysis was performed by logistic regression.

Model 1: Crude logistic regression.

Model 2: Multivariable logistic regression, adjust sex, age.

Model 3: Multivariable logistic regression, adjust sex, age, HTN, and alcohol.

*P*-value with Bonferroni correction for significance was 0.01.

**Figure 1:**
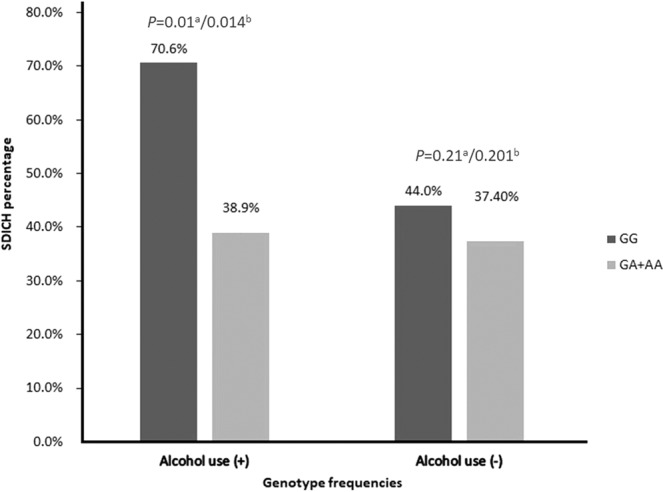
Interaction between *ALDH2* rs671 genotype and alcohol use to SDICH susceptibility. Comparisons between controls and ICH group were analysed by logistic regression under alcohol use or not. Although the interactive effect between alcohol use and rs671 genotype was borderline significant (*P* = 0.07), in those with alcohol use, the *ALDH2* rs671 GG genotype was a significant risk for SDICH compared to the rs671 GA+AA genotype (SDICH percentage: GG vs GA+AA: 70.6% vs 38.9%, OR = 0.3, 95% CI 0.1–0.8, *P* = 0.01^a^) and borderline significance (*P = *0.014^b^) while adjusting for sex and age. In contrast, the SDICH risk was similar between genotypes (*P = *0.21^a^ and *P* = 0.201^b^) in alcohol-free subjects. ^a^Crude logistic regression. ^b^Multivariable logistic regression, adjust for sex and age.

We further stratified the allelic and genotypic frequencies of *ADH1B* rs1229984, *ADH1C* rs2241894, *ALDH2* rs671, *ALDH2* rs886205, and *ALDH2* rs4648328 according to alcohol use. When stratified by alcohol use, the *ALDH2* rs671 GA genotype was significantly associated with SDICH in the alcohol use group (OR = 0.2, 95% CI: 0.1–0.7, *P* = 0.008), indicating the interaction between the *ALDH2* rs671 genotype and alcohol use. Specifically, in alcohol-free subjects, the SDICH risk was similar between genotypes. In subjects with alcohol use, SDICH was more frequently observed in individuals carrying *ALDH2* rs671 GG genotype compared to rs671 GA+AA genotype (SDICH percentage: GG vs GA+AA: 70.6% vs 38.9%, OR = 0.3, 95% CI 0.1–0.8, crude *P* = 0.01, Fig. 1), whereas this difference was not observed after multivariable adjustment. There was no association between all tested SNPs and SDICH by stratification according to the presences of hypertension and gender (data not shown). None of the alleles and genotypes in this study showed associations with hypertension (Supplementary Table S1).

**Figure 2:**
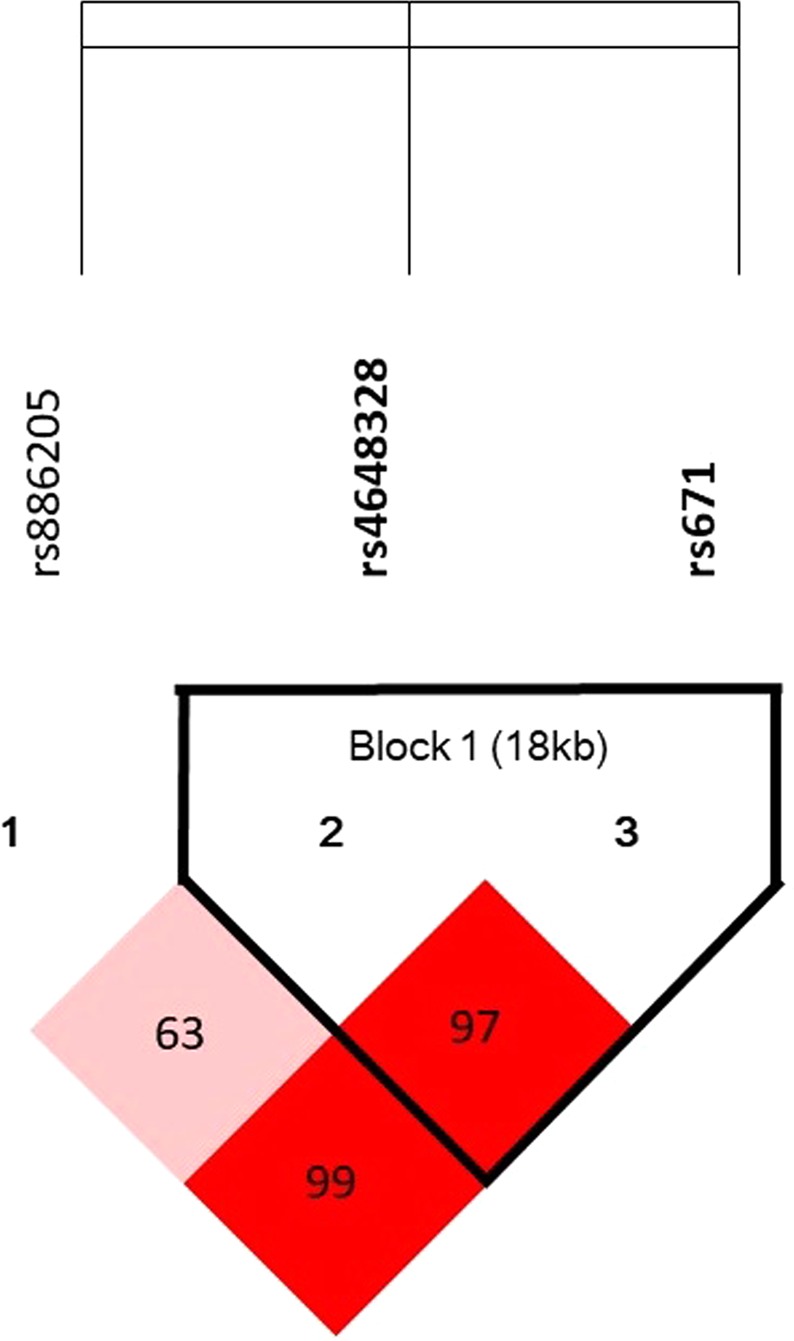
Linkage disequilibrium (LD) between the SNP markers in *ALDH2* in the Taiwanese population. Graphical representation of SNPs in Haploview linkage disequilibrium (LD) of *ALDH2* gene in SDICH patients and controls. Haploview LD coefficients D′ × 100 were generated by Haploview 4.2 and shown in each cell using the standard color scheme. D′ values of “0” indicates the independence of the examined two loci while a value of “1” demonstrates complete linkage. The strength of LD is depicted by red intensity, which moves from white to red as D′ × 100 progresses from 1 to 100. Two SNPs (rs671 and rs4648328) constitute one haplotype block that span 18 kb of *ALDH2* gene with strong linkage disequilibrium (LD), shown in bright red (D′: 0.97; r^2^: 0.11). The LD values were presented as D′: 0.99 (r^2^: 0.05) between rs671 & rs886205 and D′: 0.63 (r^2^: 0.02) between rs4648328 & rs886205 respectively.

We further characterized the *ALDH2* SNPs by linkage disequilibrium (LD) and haplotype analyses. LD analysis showed that rs4648328 and rs671 were highly correlated with each other (Fig. 2). Haplotype analysis demonstrated that the haplotype “GT” of rs671-rs4648328 was associated with SDICH (OR = 1.4; 95% CI: 1.0~1.8, *P* = 0.047, Table 4). In contrast, haplotype “AC” demonstrated protective effect on SDICH (OR = 0.6; 95% CI: 0.5~0.9, *P* = 0.005).

**Table 4:** The association between haplotypes of *ALDH2* genetic polymorphisms and the risk of spontaneously deep intracerebral hemorrhage (SDICH).

	**rs671**	**rs4648328**	Case (freq%)	Control(freq%)	OR (95% CI)	Fischer’s P
Genotypes	GG/GA/AA	CC/CT/TT				
Haplotype						
Hap1	A	C	21.5	29.8	0.6 (0.5 ~ 0.9)	0.005
Hap2	G	T	28.0	22.4	1.4 (1.0 ~ 1.8)	0.047
Hap3	G	C	50.1	47.8	1.1 (0.9 ~ 1.4)	0.452

ALDH, aldehyde dehydrogenase; CI, confidence interval; Hap, haplotype; OR, odds ratio.

## Discussion

This study, at the first time, describes the potential association between SNPs of ADH and ALDH2 with SDICH susceptibility in the Taiwanese population. Asian populations have higher incidences of SDICH with high mortality and long-term disability than Caucasians^2,3,19^. Alcohol use demonstrates the association with SDICH^7^. Alcohol is primarily metabolized by ADH and ALDH^8^. Our results support a role for *ALDH2* genetic variants in SDICH. We found that the *ALDH2* rs671 GG genotype could be a risk locus for SDICH, particularly in subjects who used alcohol, in Taiwanese population. Haplotype analysis further identified the association between haplotypes in rs671-rs4648328 of *ALDH2* and SDICH. Further large case-control cohorts in multi-ethnicities are needed to validate this association.

The rs671 is a functional SNP (Glu504Lys) in *ALDH2*^20^. Minor allele (A allele) of rs671 results in reduced ALDH2 enzymatic activity. Approximately 30% of people in Asia and 47% of those in Taiwan carrying the rs671 A allele^21–24^. In the male Japanese population, the *ALDH2* rs671 GG genotype is associated with cerebral lacunar infarcts^17^. *ALDH2* rs671 A allele are associated with coronary artery disease in Chinese patients with hypertension^16^. Moreover, *ALDH2* rs671 A allele is also associated with hypertension and cerebral amyloid angiopathy^18,25^. Although *ALDH* rs671 AA genotype may be associated with alcoholism-related hypertension^26^, our results did not detect the association between *ALDH2* rs671 and hypertension, supporting the primary effect of *ALDH2* rs671 on SDICH. *ALDH2* rs671 GG genotype tends to be a risk factor for SDICH, particularly in the group with alcohol use in Taiwanese.

*ALDH2* rs4648328, an intronic SNP, was associated with delayed alcohol metabolism in European population^27^. In our analysis, we found a potential association between rs4648328 and SDICH in the recessive and additive models. SNPs in *ALDH2* demonstrated strong LD in Indian population^27^. In addition, our study showed that rs4648328 was in LD with rs671 in Taiwanese population. The haplotype “GT” of *ALDH2* rs671-rs4648328 was associated with SDICH, whereas the haplotype “AC” demonstrated protective effect on SDICH. This study provides a baseline for future research about the role of the *ALDH2* loci in SDICH in Taiwanese population. Further large-scale investigations are needed to confirm this result.

Table 5 shows ethnicity differences in SNPs of ADH and ALDH. The genetic distributions of *ALDH2* rs671 showed strong ethnic heterogeneity. The frequencies of A allele in Taiwanese (26.3%) and East Asians (17.4%) are much higher compared to Americans (0%), Europeans (0%) and global populations (3,6%). Previous studies showed that genetic variants of *ALDH2* rs671 were associated with both alcohol flushing and alcohol use in Asian populations^28,29^. Additionally, the *ALDH2* rs671 GG genotype is associated with cerebral lacunar infarcts in the male Japanese^17^. Our study showed that rs671 GG genotype was associated with SDICH susceptibility, particularly in the alcohol use group.

**Table 5:** Minor allele frequency (MAF) in different populations.

Gene	SNP ID	MAF						
		Present study	Global^a^	East Asian^a^	South Asian^a^	American^a^	Europe^a^	Africa^a^
Sample size		N = 452	N = 5008	N = 1008	N = 987	N = 694	N = 1006	N = 1322
ALDH2	**rs671**	A/0.263	A/0.036	A/0.174	A/0.000	A/0.000	A/0.000	A/0.002
	**rs4648328**	T/0.249	T/0.200	T/0.263	T/0.210	T/0.150	T/0.159	T/0.204
	**rs886205**	A/0.141	A/0.491	A/0.156	G/0.290	G/0.310	G/0.166	A/0.223
ADH	**rs1229984**	C/0.262	T/0.159	C/0.303	T/0.020	T/0.060	T/0.029	T/0.002
	**rs2241894**	T/0.282	C/0.472	T/0.236	T/0.400	C/0.170	C/0.231	C/0.495

SNP: Single-nucleotide polymorphism; MAF: minor allele frequency.

^a^MAF data from 1000 genome information.

In addition to rs671, the MAFs of rs886205, rs1229984, and rs2241894 also greatly differ between Asian and Caucasians^30^. Table 5 showed the ethnic heterogeneous of the rs671, rs886205 rs1229984, and rs2241894 according to 1000 genome information.

The MAF T allele was present in 15.9% of rs1229984 in global population, while the rs1229984 C allele was present in 26.2% of Taiwanese and 30.3% of east Asian. (Table 5). While the ADH1B 1229984 CC genotype is predominant in East Asian population, it is rarely observed in Indian population^31^. The role of *ADH1B* rs1229984 in modulating alcohol consumption remains controversial. It has been reported that *ADH1B* rs1229984 C allele is associated with alcoholism31. However, a case-control study suggests that CC genotype of ADH1B rs1229984 may protect against alcohol dependence^32^. In our analysis, the *ADH1B* rs1229984 did not demonstrate association with alcohol consumption.

The MAF C allele was present in 47.2% of rs2241894 in global population, while the rs2241894 T allele was present in 28.2% of Taiwanese. A genome-wide association study also demonstrated an association between *ADH1C* rs2241894 and alcohol dependence in African and European Americans^14^. The MAF A allele was present in 49.1% of rs886205 in global population, while the rs886205 A allele was present in 14.1% of Taiwanese. A recent study reported that *ALDH2* rs886205 is associated with alcohol-dependent patients^33^. However, we found no associations between *ALDH2* rs886205, ADH1C rs2241894, alcohol use and SDICH in our analysis. This discrepancy may be contributed by the ethnic difference of genetic background, as well as the design of studies.

To our knowledge, this is the first study to propose that the *ALDH2* rs671 GG genotype is a risk factor for SDICH, particularly in an alcohol-using population. *ALDH2* rs671 and rs4648328 are particularly important in the interaction with alcohol use, one of the major environmental risk factors for SDICH. There are limitations to our study. First, this was a hospital-based study which may limit the generalization of our results to the whole population. Most of patients with SDICH were recruited from the Department of Neurology; these patients may demonstrate smaller haemorrhages compared to those admitted to the Department of Neurosurgery. Additionally, the relatively small sample size and gender imbalance may limit detection of potential genetic associations with SDICH. However, our results support the potential association of genetic variants in *ALDH2* rs671 GG genotype with SDICH risk in a Taiwanese population. Further studies in different populations are needed to validate our results.

### Conclusion

This study revealed a significant association between the genetic variants of *ALDH2* and SDICH susceptibility. Carrying the *ALDH2* rs671 GG genotype tends to be a risk factor for SDICH, particularly in those who use alcohol.

## Materials and Methods

### Patients and control subjects

Patients (age > 30 years old) with SDICH at the basal ganglia, thalamus, cerebellum, or brainstem were included in the study^4^. The size and location of SDICH were confirmed by brain computed tomography (CT). Patients with traumatic cerebral haemorrhage, haemorrhagic transformation of a cerebral infarct, vascular anomaly, and secondary intracranial haemorrhage (coagulopathy or hyper-perfusion syndrome) were excluded. Controls were defined as those without medical disease such as renal failure, myocardial infarction, cancer, stroke history, and neurodegenerative disease. A history of hypertension, diabetes mellitus, smoking, alcohol use, and lipid profile were collected from all participants. Alcohol use referred to the consumption of greater than 210 g of alcohol per week^34^. Smokers were defined as former or current smokers^35^.

This retrospective case-control study was approved by the Chang Gung Memorial Hospital Institution Ethics Review Board for human studies, and patients provided written informed consent prior to study participation (IRB201600775B0). All methods were performed in accordance with the relevant guidelines and regulations.

### Selection of SNP and genotyping

The cytogenetic location of *ALDH2* is 12q24. In the literature review, approximately 30% people in Asia and 47% in Taiwan were described to carry genetic variants of the A allele in *ALDH2* with reduced enzymatic activity^21–23^. We selected the *ALHD2* rs671 (G > A, missense variant Glu504Lys, exon 12), *ALDH2* rs4648328 (C > T, intron variant, intron 3), and *ALDH2* rs886205 (G > A, promoter, 5′-untranslated region) based on previous evidence of its association with alcohol dependence^26,31^. For *ADH* (cytogenetic location at 4q22), we selected *ADH1B* rs1229984 (T > C, missense variant Arg48His, exon 3) and *ADH1C* rs2241894 (A > G, synonymous variant Thr151, exon 5). *ADH1B* rs1229984 was previously investigated for its association with alcohol metabolism and alcohol drinking behaviours^32^. Additionally, *ADH1B* rs1229984, *ADH1C* rs2241894, and *ALDH2* rs671 are greatly different between Asians and Caucasians (Table 5)^30^.

Blood samples were collected for genotyping. The genomic DNA was extracted from peripheral leukocytes by using the Stratagene DNA extraction kit (La Jolla, CA, USA). Polymorphisms were genotyped using TaqMan SNP Assays in the ABI Prism 7900HT Sequence Detection System (Applied Biosystems, Foster City, CA, USA). The primer sets used for polymerase chain reaction amplification of each SNP region are as listed in Supplementary Table S2. Each SNP was checked for Hardy-Weinberg equilibrium using the standard χ^2^ test at a significance level of 0.05. Patterns of LD and haplotype analyses were evaluated using *SHEsis* Online Version (http://analysis.bio-x.cn/myAnalysis.php)^36^. Haplotypes with frequency <3% were excluded from association analysis.

### Statistical analysis and power estimation

All data analyses were performed using SAS Software (version 9.4; SAS Institute, Cary, NC, USA). Demographic data and the distributions of genotypes of SNPs were analysed by χ^2^ test, *t*-test, and univariate logistic regression. Multivariable logistic regression analyses were used to test the null hypothesis that the number of cases and controls did not differ with various genotypes of the five SNPs. Potential covariables included age, sex, hypertension, total cholesterol level, and alcohol use. Samples were stratified by alcohol use using multivariable logistic regression. All *P* values were two-tailed. While considering Bonferroni correction, the significance level was set to 0.01. Given the observed allele frequency in the present case-control study, at the 0.01 significance level, we had power greater than 0.8 to identify an association of the genetic variant with SDICH susceptibility when the per-allele genetic effect was greater than an odds ratio of 1.8 for rs886205 and 1.7 for the rest of the SNPs.

## Supplementary information

Supplementary Tables.
