# Supplementary material for: Association of alcohol dehydrogenase and aldehyde dehydrogenase Polymorphism with Spontaneous Deep Intracerebral Haemorrhage in the Taiwan population
Source: Sci Rep. 2020 Feb 27;10:3641. doi: 10.1038/s41598-020-60567-5 (PMC7046678; doi:10.1038/s41598-020-60567-5)
Supplement: Supplementary file 1 — Supplementary Tables. [file 41598_2020_60567_MOESM1_ESM.pdf]

Association of alcohol dehydrogenase and aldehyde dehydrogenase Polymorphism with Spontaneous Deep Intracerebral Haemorrhage in the Taiwan population

Yu-Hua Huang [1], Kuo-Hsuan Chang[1], Yun-Shien Lee[2,3], Chiung-Mei Chen[1], Yi-Chun Chen[1*]

[1] Department of Neurology, Chang Gung Memorial Hospital Linkou Medical Center and College of Medicine, Chang-Gung University, Taoyuan, 333, Taiwan

[2] Department of Biotechnology, Ming Chuan University, Taoyuan, 333, Taiwan

[3] Genomic Medicine Research Core Laboratory, Chang Gung Memorial Hospital, Taoyuan, 333, Taiwan

[*] Corresponding author

Yi-Chun Chen

E-mail: asd108@adm.cgmh.org.tw (YCC)

Supplementary Table S1. Genotype frequencies of the SNPs and their associations with risk of spontaneously deep intracerebral hemorrhage (SDICH) and hypertension

Gene	SNP ID	Genotype	Frequencies		Model 1	Model 2	Model 3
			HTN cases (%)	Control (%)	OR (95% CI), *P* value	*P* value	*P* value
ALDH2	**rs671**	GG	174(60.0)	77(47.8)	1		
		GA	95(32.8)	68(42.2)	0.6(0.4-0.9), 0.022	0.015	0.038
		AA	21(7.2)	16(9.9)	0.6(0.3-1.2), 0.130	0.073	0.142
		Dominant model			0.6(0.4-0.9), 0.013	0.007	0.024
		Additive model			1.4(1.1-1.9), 0.019	0.009	0.031
		Recessive model			0.7(0.4-1.4), 0.319	0.214	0.356
	**rs4648328**	CC	160(55.2)	98(60.9)			
		CT	104(35.9)	57(35.4)	0.595	0.541	0.648
		TT	26(9.0)	6(3.7)	0.038	0.039	0.066
		Dominant model			1.3(0.9-1.9),0.242	0.220	0.325
		Additive model			0.8(0.5-1.0),0.077	0.071	0.126
		Recessive model			2.5(1.0-6.3),0.044	0.047	0.074
	**rs886205**	GG	214(73.8)	118(73.3)			
		GA	71(24.5)	40(24.8)	0.925	0.962	0.983
		AA	5(1.7)	3(1.9)	0.908	0.873	0.876
		Dominant model			1.0(0.6-1.5),0.908	0.992	0.956

	Additive model			1.0(0.7-1.5),0.893	0.972	0.927
	Recessive model			0.9(0.2-3.9),0.914	0.870	0.877
**rs1229984**	TT	155(53.5)	86(53.4)			
	TC	118(40.7)	66(41.0)	0.969	0.985	0.997
	CC	17(5.9)	9(5.6)	0.914	0.844	0.842
	Dominant model			1.0(0.7-1.5),0.995	0.944	0.960
	Additive model			1.0(0.7-1.4),0.968	0.893	0.905
	Recessive model			1.1(0.5-2.4),0.906	0.844	0.838
**rs2241894**	CC	151(52.1)	76(47.2)			
	CT	123(42.4)	70(43.5)	0.550	0.641	0.605
	TT	16(5.5)	15(9.3)	0.107	0.072	0.066
	Dominant model			0.8(0.6-1.2),0.323	0.354	0.325
	Additive model			1.3(0.9-1.7),0.155	0.144	0.128
	Recessive model			0.6(0.3-1.2),0.131	0.082	0.077

Model 1: Crude logistic regression

Model 2: Multivariable logistic regression, adjust sex, age

Model 3: Multivariable logistic regression, adjust sex, age, and alcohol

Supplementary Table S2. Primers for Genotyping of Single Nucleotides Polymorphisms (SNPs) Using the TaqMan SNP Assays

Gene	SNP	Clone ID	Forward Primers (F)and VIC-Probe	Reverse Primer (R)and FAM-Probe
ADH1C	rs2241894	C_2688508_10	VIC-TGGCCACTGCATTCTCATCCACCACCGTGTACTGGGAGAAGGTGCTGACGC	FAM-TGGCCACTGCATTCTCATCCACCACTGTGTACTGGGAGAAGGTGCTGACGC
ADH1B	rs1229984	C_2688467_20	VIC-GCCACTAACCACGTGGTCATCTGTGCGACAGATTCCTACAGCCACCATCTA	FAM-GCCACTAACCACGTGGTCATCTGTGTGACAGATTCCTACAGCCACCATCTA
ALDH2	rs671	C_11703892_10	VIC-CGAGTACGGGCTGCAGGCATACACTAAAGTGAAAACTGTGAGTGTGGGACC	FAM-CGAGTACGGGCTGCAGGCATACACTGAAGTGAAAACTGTGAGTGTGGGACC
	rs4648328	C_28028878_10	VIC-AGCTCCACGCTGGTCTCTATCCCAGCACCTGGATATGGTAGAGAGCAGAAG	FAM-AGCTCCACGCTGGTCTCTATCCCAGTACCTGGATATGGTAGAGAGCAGAAG
	rs886205	C_2547557_30	VIC-CTCTTCACCTGGAGCATCAGCCGGGAAGGTCAGGGTCGCCCTGGCTCGGGC	FAM-CTCTTCACCTGGAGCATCAGCCGGGGAGGTCAGGGTCGCCCTGGCTCGGGC
